# Zinc and curcumin lower arylsulfatses and some metabolic parameters in streptozotocin-induced diabetes

**DOI:** 10.1186/s40200-017-0293-7

**Published:** 2017-03-14

**Authors:** Mahmoud Balbaa, Marwa El-Zeftawy, Nabil Taha, Abdel-Wahab Mandour

**Affiliations:** 1 Departments of Biochemistry, Faculty of Science, Alexandria University, Moharram Bey, 21511 Alexandria, Egypt; 2 Edfina Faculty of Veterinary Medicine, Alexandria University, Alexandria, Egypt

## Abstract

In rats with induced diabetes, Zinc and curcumin treatment showed a significant increase of catalase and a significant decrease of glucose, lipid profile components and arylsulphatases activity compared to the untreated rats. We suggest that dietary zinc and curcumin are promising protective agents for reducing the metabolic defect of diabetes.

## Dear Editor;

Diabetes mellitus is a metabolic disease and the pathogenesis of diabetes mellitus is implicated in the oxidative stress and the generation of superoxide free radicals [1]. Various small molecules have been investigated for their ability to ameliorate the diabetes. One such molecule is curcumin (Cur) that has various health beneficial properties such as anti-inflammatory, anticarcinogenic, antiviral, hypolipidemic and antiinfectious activities [2, 3]. The 2nd molecule is the zinc (Zn) salt as an essential trace element. The disturbances of its homeostasis seem to be associated not only with diabetes, but also with others [4]. Recently, it was reported that the lysosomal enzymes arylsulfatases were significantly changed in experimental diabetes [5]. In fact, the treatment of diabetes through food sources is valuable around the world. Therefore, the present study is undertaken to throw the light on the effect of Zn and Cur on rats with experimental diabetes rats through studying the effect on some lipid components and arylsulfatases as important parameters, which are implicated in different biological functions.

Male albino rats (120–160 g Bwt) were kept on a balanced ration with water *ad libitum* for acclimatization. Experimental diabetes was induced in overnight fasted rats by intraperitoneal injection of a single dose of streptozotocin (STZ) as 60 mg/kg Bwt. Rats with a serum glucose level 218 mg/dl were considered as rats with diabetes. The daily intake of Zn sulfate (100 mg/kg Bwt) and was administrated orally in non-ionized water for 60 days. Cur was suspended in saline and administrated orally by a gavage 80 mg/kg Bwt Cur suspended daily in saline for 60 days. The rats were grouped randomly into eight equal groups as the non-diabetic (control group), the diabetic group, Zn non-diabetic group, Zn diabetic group, Cur non-diabetic group, Cur diabetic group. The last two groups that belong to Zn and Cur non-diabetic and diabetic group received 50 and 40 mg/kg Bwt, respectively as a daily for 60 days. The animals were deprived of food overnight and sacrificed by decapitation. Blood was collected from the eye canthus in tubes containing potassium oxalate and sodium fluoride mixture for estimation of plasma glucose (PG). Liver or pancreas tissues were weighed, homogenized in10 mM Tris HCl buffer, pH 7.0 and centrifuged at 4000 rpm for 15 min at 4 °C. The clear supernatant was obtained to measure the activities of catalase [6], total protein [7] and both arylsulfatase A (ASA) and arylsulfatase B (ASB) [8]. ASA and ASB were fractionated by DEAE-cellulose chromatography as described previously [8]. Insulin was assayed in the homogenate of pancreas of all groups according to instruction of Sigma-Aldrich insulin ELISA kit. Serum total cholesterol (STC) was determined by cholesterol oxidase and peroxidase [9]. Serum triacylglycerol (STG) was determined as described previously [10]. Serum LDL cholesterol (SLDL-c) and HDL cholesterol (SHDL-c) levels were estimated as that described previously [11]. The obtained data during the experimental period were statistically analyzed by the paired sample T-test (SPSS version 16).

A significant increase of plasma glucose was noted after one and 60 days of STZ injection. Oral administration of Zn, Cur and a combination of both showed a significant effect on blood glucose levels (1.9 fold-increase). Similar results were obtained by the effect of Cur, whereas an increase of plasma sugar level of 4.7 folds in day one was changed to be only 1.7 fold after 60 days of STZ-treatment. Interestingly, on treating the rats by both Zn and Cur, the noticed significant change of plasma sugar level in day one was changed to be non-significant at 60 days of STZ-treatment (Table 1). A reversed tendency to that of plasma glucose was shown on measuring the insulin content of pancreatic homogenate of animals from the different groups. The rats with diabetes showed a significant decrease (4.4 fold) in insulin compared to control (*p* < 0.001). Insulin level showed a non-significant change after treatment with Zn and Cur. In addition, a significant increase in the levels of serum total cholesterol (STC) and triacylglycerol (STG) in the diabetic groups was noticed in comparison to the non-diabetic groups. Comparison of the diabetic groups versus the non-diabetic groups showed a significant decrease in both SHDL-c and SLDL-c levels in the diabetic groups and a significant increase after treatment with Zn and Cur (Table 1). For studying the effect of Zn and Cur on free radical production, the activity of catalase was measured in both plasma and liver. It presented a significant decrease in diabetic compared to control rats. The effect of Zn and Cur showed a significant increase of the specific activity in serum and hepatic catalase compared to STZ-induced diabetic rats (*p* < 0.05). Furthermore, there is a significant increase in the specific activities of ASA and ASB in both serum and liver in the rats of the diabetic group compared to control (Table 2). Zn and Cur administration decrease it significantly compared to the diabetic group (*p* < 0.05). The significant increase of catalase by Zn may be attributed to the competition of Zn to both iron and copper for binding to cell membranes and thus decreasing the production of OH^-^ group [12]. This group in turn stimulates the peroxidation of membrane lipids and hence the outflow of lysosomal constituents into cytosol [13]. The role of combination of both Zn and Cur in diabetes showed a highly significant effective result than the use of Zn or Cur alone. Taken together, we suggest that Zn and Cur have an effective and a protective role against the effect of diabetes—produced radicals on lysosomes. Dietary Zn and Cur are promising protective agents with a potential therapeutic approach to diabetes.

**Table 1:** Effect of Zn and curcumin on Lipid Profile distribution

Groups	Plasma glucose (PG, mg/dl)	Serum total cholesterol	Serum triacylglycerol	Serum LDL	Serum HDL
Control	111.84 ± 2.56	50.25 ± 1.54	37.2 ± 1.73	63.75 ± 3.20	106.56 ± 3.04
Diabetic	381.81 ± 15.12^**^	137.02 ± 7.04^**^	635.63 ± 29.57^**^	50.71 ± 6.22^**^	41.79 ± 1.38^**^
Zn non-diabetic	73.61 ± 2.21^*^	32.67 ± 1.31^*^	37.9 ± 1.01	72.16 ± 3.40	79.24 ± 2.83^*^
Zn-treated diabetic	138.59 ± 7.81^*^	60.34 ± 3.67^*^	116.40 ± 3.54^*^	61.98 ± 4.44	99.04 ± 3.19
Cur non-diabetic	74.47 ± 2.99^*^	47.49 ± 1.45	23.95 ± 0.86^*^	56.70 ± 2.24	99.99 ± 2.19
Cur-treated diabetic	124.88 ± 4.36^*^	59.13 ± 3.07^*^	30.95 ± 1.39^*^	28.51 ± 3.60*	82.61 ± 3.40^*^
Zn and Cur non-diabetic	111.08 ± 4.39	47.15 ± 1.60	34.53 ± 1.66	58.09 ± 3.01	98.33 ± 2.40
Zn and Cur- treated diabetic	118.22 ± 4.90	51.47 ± 1.37	41.3 ± 2.30	38.88 ± 2.56^*^	82.89 ± 2.50^*^

The mean values of the serum level (mg/dl) of total cholesterol, triacylglycerol and the lipoproteins LDL and HDL in control, diabetic, Zn- and curcumin-treated diabetic groups. The values are the means of 12 rats ± SE. Significance: *p < 0.05 and **p < 0.001 compared to control

**Table 2:** Effect of Zn and Cur on the specific activity of catalase (units/mg protein) and arylsulfatases ASA and ASB (nmol product/h/mg protein)

Groups	Serum Catalase​(x 10^4^)	Hepatic Catalase​(x10^4^)	Serum ASA	Hepatic ASA	Serum ASB	Hepatic ASB
Control	1.09 ± 0.05	28.5 ± 1.4	12.01 ± 0.74	226.78 ± 10.25	23.28 ± 1.50	505.82 ± 23.03
Diabetic	0.49 ± 0.03^**^	4.4 ± 0.4^**^	25.58 ± 0.89^**^	555.36 ± 52.49^**^	34.24 ± 1.90^*^	627.79 ± 53.18^**^
Zn non-diabetic	1.00 ± 0.03	36.4 ± 2.8^*^	15.25 ± 0.81	268.04 ± 6.78^*^	34.69 ± 1.62^*^	531.61 ± 17.67^*^
Zn-treated diabetic	0.97 ± 0.03	34.1 ± 1.5^*^	9.53 ± 0.78^*^	214.66 ± 3.99^*^	21.42 ± 1.02	408.94 ± 15.37^*^
Cur non-diabetic	0.88 ± 0.03	36.9 ± 2.0^*^	14.86 ± 0.51	261.32 ± 8.47^*^	33.26 ± 2.08^*^	604.90 ± 11.17^*^
Cur-treated diabetic	1.03 ± 0.03	47.1 ± 1.9^*^	11.13 ± 0.66	260.33 ± 10.70^*^	32.87 ± 1.96^*^	431.83 ± 22.81^*^
Zn and Cur non-diabetic	1.01 ± 0.03	35.8 ± 1.5^*^	12.46 ± 0.23	174.60 ± 10.17^*^	31.10 ± 1.11^*^	501.37 ± 17.75
Zn and Cur-treated diabetic	1.00 ± 0.02	36.2 ± 2.5^*^	9.32 ± 0.40^*^	150.59 ± 11.39^*^	20.28 ± 1.14	333.67 ± 22.74^*^

The values are the means of 12 rats ± SE. Significance: *p < 0.05 and **p < 0.001 compared to control

## Abbreviations

ASA: Arylsulfatase A
ASB: Arylsulfatase B
Bwt: Body weight
Cur: Curcumin
PG: Plasma glucose
SHDL-c: Serum high density lipoprotein cholesterol
SLDL-c: Serum low density lipoprotein cholesterol
STC: Serum total cholesterol
STG: Serum total triacylglycerol
STZ: Streptozotocin
